# Generation of Human Blood Vessel and Vascularized Cerebral Organoids

**DOI:** 10.21769/BioProtoc.4870

**Published:** 2023-11-05

**Authors:** Xin-Yao Sun, Xiang-Chun Ju, Hong-Fang Zhao, Zhi-Wen You, Run-Run Han, Zhen-Ge Luo

**Affiliations:** 1 School of Life Science and Technology, ShanghaiTech University, Shanghai, China; 2 Institute of Neuroscience, Center for Excellence in Brain Science and Intelligence Technology, Chinese Academy of Sciences, Shanghai, China; 3 Okinawa Institute of Science and Technology, Onna-son, Japan; 4 Shanghai Institute of Nutrition and Health, University of Chinese Academy of Sciences, Chinese Academy of Sciences, Shanghai, China; 5 Cell Differentiation and Apoptosis of the Chinese Ministry of Education, Shanghai Jiao Tong University School of Medicine, Shanghai, China; 6 Division of Stem Cell Biology, Institute for Genetic Medicine, Hokkaido University, Sapporo, Hokkaido, Japan

**Keywords:** Blood vessel organoids, Cerebral organoids, Neural organoids, Vascularized brain organoids, Human embryonic stem cells

## Abstract

Brain organoids have been widely used to study diseases and the development of the nervous system. Many reports have investigated the application of brain organoids, but most of these models lack vascular structures, which play essential roles in brain development and neurological diseases. The brain and blood vessels originate from two different germ layers, making it difficult to induce vascularized brain organoids in vitro. We developed this protocol to generate brain-specific blood vessel and cerebral organoids and then fused them at a specific developmental time point. The fused cerebral organoids exhibited robust vascular network-like structures, which allows simulating the in vivo developmental processes of the brain for further applications in various neurological diseases.

Key Features

• Culturing vascularized brain organoids using human embryonic stem cells (hESCs).

• The new approach generates not only neural cells and vessel-like networks but also brain-resident microglia immune cells in a single organoid.

**Graphical overview**


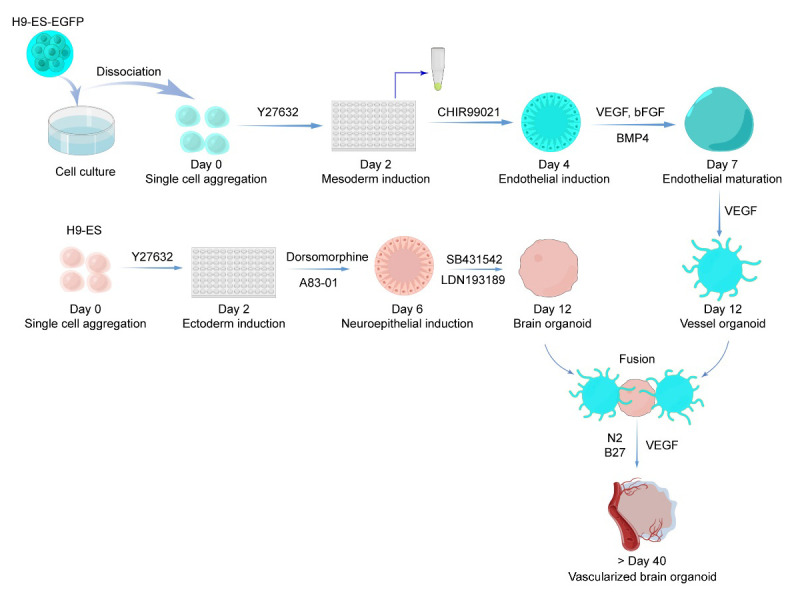


**Workflow and timeline for vessel organoid and vascularized brain organoid generation.** (By Figdraw, ID: RTIURffccf)

## Background

As stem-cell-derived 3D-microtissue, brain organoids have been developed to model developmental programs of the human fetal brain and recapitulate developmental, psychiatric, and degenerative brain diseases. The wide application of brain organoids improves our understanding of the developmental process of the human brain (Lancaster and Knoblich, 2014; Di Lullo and Kriegstein, 2017; Amin and Paşca, 2018; Benito-Kwiecinski, 2021). However, lack of a neurovascular system, which plays essential roles in oxygen supply, neurogenesis regulation, and functional application, limits the applications of brain organoids (Tata et al., 2016).

Blood vessels originate from the mesoderm and develop via sequential vasculogenesis and angiogenesis processes, which invade the neuroepithelial regions and form the brain vascular network (Lee et al., 2009). A self-organizing human blood vessel organoid model has been reported and applied to the study of diabetic vasculopathy (Wimmer et al., 2019). However, because the blood vessels and neurons in the brain are derived from two different germ layers, it has been challenging to generate vascularized brain organoids. Several studies have attempted to induce vascular structures in cerebral brain organoids, including co-cultured brain organoids with endothelial cells (ECs) or their progenitors (Mansour et al., 2018; Pham et al., 2018; Cakir et al., 2019; Shi et al., 2020; Wörsdörfer et al., 2020). These reports provide various ideas for further improvement in this field.

We recently developed an induction approach for brain-specific vascular organoids cultured in the medium containing neurotrophic factors at the maturation stage to obtain vessel organoids with cerebrovascular characteristics (Sun et al., 2022). Although the generation of vascularized neural organoids by assembling neural and mesenchymal aggregates has been reported (Wörsdörfer et al., 2020), we showed more abundant cell types in the vessel organoids, including a substantial number of microglia cells in the fusion vascularized organoids, which enabled better recapitulation of the brain characteristics in vivo. This vessel organoid model showed a variety of cell types similar to those observed in vivo. The mature vessel organoids exhibited robust vasculatures that can invade the brain organoids when wrapped in Matrigel. This approach also induced microglial cells along with other types of vascular cells. The fusion vascularized brain organoids induced by this protocol accurately simulate the structure and function of brain vessels. This protocol provides a platform for studying interactions between neuronal and non-neuronal components during brain development and function.

## Materials and reagents

**Biological materials**

1. H9 embryonic stem cell (H9-ESC, human) (WiCell, WA09)
2. H9-EGFP (generated in house)

**Reagents**

1. mTeSR1 (STEMCELL Technologies, catalog number: 85850)
2. ReLeSR^TM^ (STEMCELL Technologies, catalog number: 05872)
3. Accutase (STEMCELL Technologies, catalog number: 07920)
4. Y27632 (STEMCELL Technologies, catalog number: 72304)
5. Human Recombinant bFGF (STEMCELL Technologies, catalog number: 78003)
6. Human Recombinant VEGF (STEMCELL Technologies, catalog number: 78073)
7. Human Recombinant BMP4 (R&D, catalog number: 314-BP-050)
8. DMEM/F12 (Life/Invitrogen, catalog number: 11330032)
9. Knockout serum replacement (KSR) (Gibco, catalog number: 10828028)
10. MEM-NEAA (Gibco, catalog number: 11140050)
11. Glutamax (Gibco, catalog number: 35050061)
12. β-mercaptoethanol (Sigma-Aldrich, catalog number: M3148)
13. Dorsomorphine (Tocris, 3093/10)
14. A83-01 (Tocris, catalog number: 2939/10)
15. Neurobasal (Life/Invitrogen, catalog number: 21103049)
16. N2 supplement (Life/Invitrogen, catalog number: 17502048)
17. B27 supplement (Life/Invitrogen, catalog number: 17504044)
18. B27 supplement without vitamin A (Life/Invitrogen, catalog number: 12587010)
19. Insulin (Sigma-Aldrich, catalog number: I9278)
20. Heparin (Sigma-Aldrich, catalog number: H3393)
21. Lipidure^®^-CM5206 (NOF CORPORATION, catalog number: CM5206)
22. Antibiotic-Antimycotic (Gibco, catalog number: 15240096)
23. Matrigel hESC-qualified matrix (BD-Biocoat, catalog number: 354277)
24. Matrigel growth factor reduced (BD-Biocoat, catalog number: 354230)
25. STEMdiff APEL2 medium (STEMCELL Technologies, catalog number: 05270)
26. Endothelial cell growth medium MV2 (Promocell, catalog number: C-22022)
27. CHIR99021 (Selleck, catalog number: S1263)
28. LDN-193189 2HCl (Selleck, catalog number: S7505)
29. SB431542 (Selleck, catalog number: S1067)
30. DPBS (Life/Invitrogen, catalog number: 14190144)
31. Mouse anti-CD31 (Abcam, catalog number: ab9498, 1:500)
32. Goat anti-DCX (Santa Cruz, catalog number: sc-8066, 1:200)
33. Chicken anti-GFP (Aves Lab, SKU number: GFP-1020, 1:1,000)
34. Rabbit anti-IBA1 (Wako, catalog number: 019-19741, 1:500)
35. DAPI fluorochrome (Beyotime, catalog number: C1002)
36. Paraformaldehyde (PFA) (Sigma-Aldrich, catalog number: 158127)
37. Triton X-100 (Sigma-Aldrich, catalog number: T8787)
38. Bovine serum albumin (Sigma-Aldrich, catalog number: A9418)
39. Sucrose (Sinopharm, catalog number: 10021418)
40. Antigen retrieval buffer (10 mM citric acid, pH 6.0) (Sigma-Aldrich, catalog number: 1159047)
41. Optimal cutting temperature (OCT) compound (Sakura, catalog number: 4583)
42. Aggregation medium (see Recipes)
43. Mesoderm induction medium (see Recipes)
44. Endothelial induction medium (see Recipes)
45. Endothelial maturation medium (see Recipes)
46. Expansion medium (see Recipes)
47. Maturation medium (see Recipes)
48. Ectoderm induction medium (see Recipes)
49. Neuroepithelium induction medium (see Recipes)

## Recipes

*Note: The basal medium could be stored at -20 °C for six months and thawed immediately before use. However, as presented in the following recipes, all culture media can be stored for up to two weeks at 4 °C. The small molecules and growth factors (Y27632, CHIR99021, VEGF, BMP4, bFGF, dorsomorphine, A83-01, LDN193189, and SB431542) should be added immediately before use.*

1. **Aggregation medium**
  **Table:** 

Reagents	Final concentration	Volume
mTeSR1		50 mL
Y27632	10 μM	50 μL
2. **Mesoderm induction medium**
  **Table:** 

Reagents	Final concentration	Volume
APEL2		10 mL
CHIR99021	6 μM	12 μL
3. **Endothelial induction medium**
  **Table:** 

Reagents	Final concentration	Volume
APEL2		20 mL
VEGF	50 ng/mL	20 μL
BMP4	25 ng/mL	10 μL
bFGF	10 ng/mL	4 μL
4. **Endothelial maturation medium**
  **Table:** 

Reagents	Final concentration	Volume
MV2		50 mL
VEGF	50 ng/mL	50 μL
5. **Expansion medium**
  **Table:** 

Reagents	Final concentration	Volume
DMEM/F12		500 mL
Neurobasal		500 mL
N2 supplement		5 mL
B27 supplement without vitamin A		10 mL
β-mercaptoethanol		3.5 μL
Insulin		250 μL
Glutamax		10 mL
MEM-NEAA		5 mL
Antibiotic-Antimycotic		10 mL
VEGF	20 ng/mL
6. **Maturation medium**
  **Table:** 

Reagents	Final concentration	Volume
DMEM/F12		500 mL
Neurobasal		500 mL
N2 supplement		5 mL
B27 supplement		10 mL
β-mercaptoethanol		3.5 μL
Insulin		250 μL
Glutamax		10 mL
MEM-NEAA		5 mL
Antibiotic-Antimycotic		10 mL
VEGF	20 ng/mL
*Note: The human recombinant VEGF is added immediately before use in the expansion and maturation mediums, with the final concentration of 20 ng/mL.*
7. **Ectoderm induction medium**
  **Table:** 

Reagents	Final concentration	Volume
DMEM/F12		400 mL
KSR		100 mL
β-mercaptoethanol		3.5 μL
Glutamax		5 mL
MEM-NEAA		5 mL
Dorsomorphine	2.5 μM	
A83-01	2 μM
8. **Neuroepithelium induction medium**
  **Table:** 

Reagents	Final concentration	Volume
DMEM/F12		500 mL
N2 supplement		5 mL
Glutamax		5 mL
MEM-NEAA		5 mL
Heparin	1 μg/mL	
SB431542	10 μM	
LDN193189 2HCl	200 nM

**Laboratory supplies**

1. Cell culture 6-well plates (Corning, catalog number: 3516)
2. V-bottom 96-well plates (Thermo, catalog number: 277143)
3. Cell culture 60 mm dishes (Corning, catalog number: 430166)
4. Cell culture 35 mm dishes (Corning, catalog number: 430165)
5. Pasteur pipettes, 3 mL (Nest, catalog number: 318314)
6. Serological pipette, 5 mL (Corning, catalog number: 4487)
7. Serological pipette, 10 mL (Corning, catalog number: 4492)
8. Serological pipette, 25 mL (Corning, catalog number: 4251)
9. Sterile tips (Axygen)
10. Sterile 15 mL polypropylene centrifuge tubes (Corning, catalog number: 430790)
11. Sterile 50 mL polypropylene centrifuge tubes (Corning, catalog number: 430828)

## Equipment

1. Olympus FV3000 confocal laser scanning microscope
2. Olympus CKX53 microscope
3. Water bath (Jinghong, model: XMTD-8222)
4. Biosafety cabinet (Thermo, model: 1300 SERIES A2)
5. CO_2_ constant-temperature incubator (Thermo, model: HERAcell 150i)
6. Pipette sets (Eppendorf)
7. Table shaker (SCILOGEX SLC-O3000-S)
8. Centrifuge (Eppendorf, model: 5804R)
9. Cryostat (Thermo, model: NX50)
10. Freezers (Panasonic, model: Haier)

## Software

1. Fiji/ImageJ (ImageJ 1.52e, https://fiji.sc, access date 07/11/2018)
2. OLYMPUS FV31S-SW
3. Figdraw (https://www.figdraw.com/static/index.html#/, access date 02/14/2022)

## Procedure

- **H9-EGFP and H9-ES cell culture**
  The identity of the H9 human embryonic stem (H9-hES) cell line was confirmed by short tandem repeat (STR) profiling (performed by APPLIED CELL). The cells were tested for mycoplasma, and the result was negative. The H9 cell line stably expressing H9-EGFP was generated by introducing a CAG-EGFP DNA fragment into the genome locus ROSAβgeo26 (ROSA26) using the clustered regularly interspaced short palindromic repeats-associated protein 9 (CRISPR/Cas9) method.
*Note: The experiments published in the original research paper and described here were performed using the H9 cell line purchased from APPLIED CELL. Unfortunately, the company no longer provides this cell line. We suggest directly ordering the H9 cell line (WA09) from WiCell Research Institute (Madison, WI, USA) or using an alternative cell line, such as an iPS cell line from WiCell (UCSD093i-1-11), which we have tested yielding similar results. In addition, mycoplasma testing is crucial, because cells infected with mycoplasma can have their growth and differentiation affected; for the testing protocol, refer to this latest study by Siegl et al. (2023).*
  1. Coat 6-well plates using hESC-qualified Matrigel
    - Thaw the frozen Matrigel stock solution in the fridge (4 °C) overnight and place the vial on ice to avoid gel formation.
    - To prepare the working solution, dilute Matrigel stock solution by adding cold (4 °C) DMEM/F12 into the vial on ice and keep pipetting until the material is evenly dispersed.
    - Add 1 mL of Matrigel working solution per well and distribute the solution gently to cover the bottom surface. Then, incubate the Matrigel-coated plate for 1 h in the cell incubator (37 °C).
*Note: The best dilution ratio of the Matrigel stock solution is based on the batch number (LOT number); please check on the official Corning website. Before coating, Matrigel stock solution dilution must be kept on ice. Otherwise, it will irreversibly solidify quickly when the highly concentrated Matrigel stock solution is placed at room temperature (RT). If you need to pipette the concentrated Matrigel solution, freeze the tips in the freezer ahead of time to avoid the clogging of Matrigel in the tip. The lot numbers we have tested are 2088003/2095003/2109003/2116003/2158003/2160996/2165002/2249003/2250003/2319003 (e.g., visit the website* *https://www.corning.com/asean/en/products/life-sciences/resource-library.html?productNumber=354277&lotNumber=* , *input the lot number 2088003, and click on* Download certificate. *The “dilution factor: 310 μL” implies that 310 μL of Matrigel is diluted by the mTeSR medium up to 25 mL for the final concentration.)*
  2. Cell thawing
    - Add 10 μM of Y27632 (Rock inhibitor) to the embryonic stem cells (ESC) culture medium (mTeSR) to prevent cell death and warm the medium at 37 °C for 30 min (the warming time depends on the volume of the medium).
    - Remove the cryotube from liquid nitrogen and quickly thaw the cells in a 37 °C water bath with only a small ice pellet remaining.
    - Gently add 1 mL of warm mTeSR medium in a dropwise manner to the cells to avoid a sudden change in the osmolarity of the freezing solution around the cells and improve recovery; then, transfer the cells into a 15 mL conical tube with fresh mTeSR medium (3 mL per cryovial) and centrifuge at 200× *g* for 2 min.
*Note: A sudden change in the osmolarity of the cryoprotectant around the cells may cause a rapid stream of water across the membranes of the cells, which could stress the cells and make them more prone to dying. Avoiding this stressful operation improves cell survival and recovery.*
    - Remove the supernatant medium and resuspend the cells in fresh mTeSR medium containing rho-associated protein kinase (ROCK) inhibitor (2–3 mL per culture well for the 6-well plate) using a 1,000 μL tip by pipetting 2–3 times as gently as possible.
    - Aspirate the coating Matrigel solution and transfer the cell suspension into the coated well.
*Note: When warming the medium, the use of a water bath with circulating water will shorten the time. The experimenter should check the warmed medium by gripping the bottle with a hand and avoid using the cold medium. When resuspending cells, pipette as few times as possible. Do not use sharp tips (e.g., 200 μL tip) to pipette cells, as this will cause severe cell death. Y27632 is added into the medium only on the first day of cell resuscitation.*
  3. Cell maintenance
    Aspirate the medium from the 6-well plate that contains human ESC (hESC) colonies and add 2 mL of fresh culture medium (mTeSR with 4 ng/mL bFGF) on the following day when the cells are well attached to the well. Replace the culture medium daily.
*Note: The culture medium can be changed every other day if only a few dead cells exist. Check the status of stem cells based on the colony and cell morphology (Wakui et al., 2017), e.g., high nucleus/cytoplasm ratio of cells, flat and well-defined edge of colonies (see also step B1).*
  4. Cell passage
    - Prepare a Matrigel-coated plate as described above before starting the cell passage.
    - Aspirate the mTeSR medium from the well containing the hESC colonies and wash with 1× DPBS.
    - Replace the DPBS with 1 mL of ReLeSR^TM^ solution for each well of the 6-well plate and incubate for 30 s. Then, aspirate the ReleSR^TM^ quickly and place the cell plate in the incubator for another 3 min.
    - Take out the culture plate and gently add the warm mTeSR medium along the wall of the wells.
    - Gently tap the sides of the culture plate to detach the cell colonies from the bottom of the wells into the fresh medium. Then, dissociate detached colonies into small clumps by gentle pipetting using a 1,000 μL tip.
    - Seed the cell clumps at a split ratio of approximately 1:10 in a new Matrigel-coated well and return the plate to the incubator. Tilt the plate several times in both the horizontal and vertical directions to evenly spread the cell clumps.
    - Change the culture medium daily until 80%–90% confluence is achieved for organoid generation.
*Note: Use ReleSR^TM^ as the dissociation reagent to detach the cell colonies rather than dissociating the colonies into single cells. Generally, cells should be passaged before the clones adhere together. During cell passage and maintenance, basic fibroblast growth factor (bFGF) should be added into the culture medium right just before use, as the bFGF activity may markedly decline in the medium if added too early.*
- **Blood vessel organoid (VOr) generation (schematic view shown in Figure 1)**
  **Figure 1.**
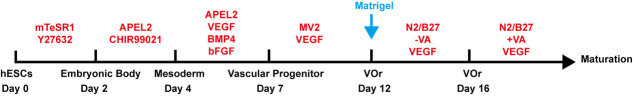
Schematic procedure of generating vessel organoids (VOrs) from human embryonic stem cells (hESCs)
  1. Blood vessel organoid aggregation, day 0
    - Culture H9-EGFP cells in a new 6-well plate as described in section A; the EGFP tag marks the vascular part in the subsequent fusion process. When the cell density reaches approximately 80% confluence and shows good growth status (flat and well-defined edges of colonies and high ratio of nucleus to cytoplasm in cells) (Figure 2A), the stem cells are ready for organoid generation.
      **Figure 2.**
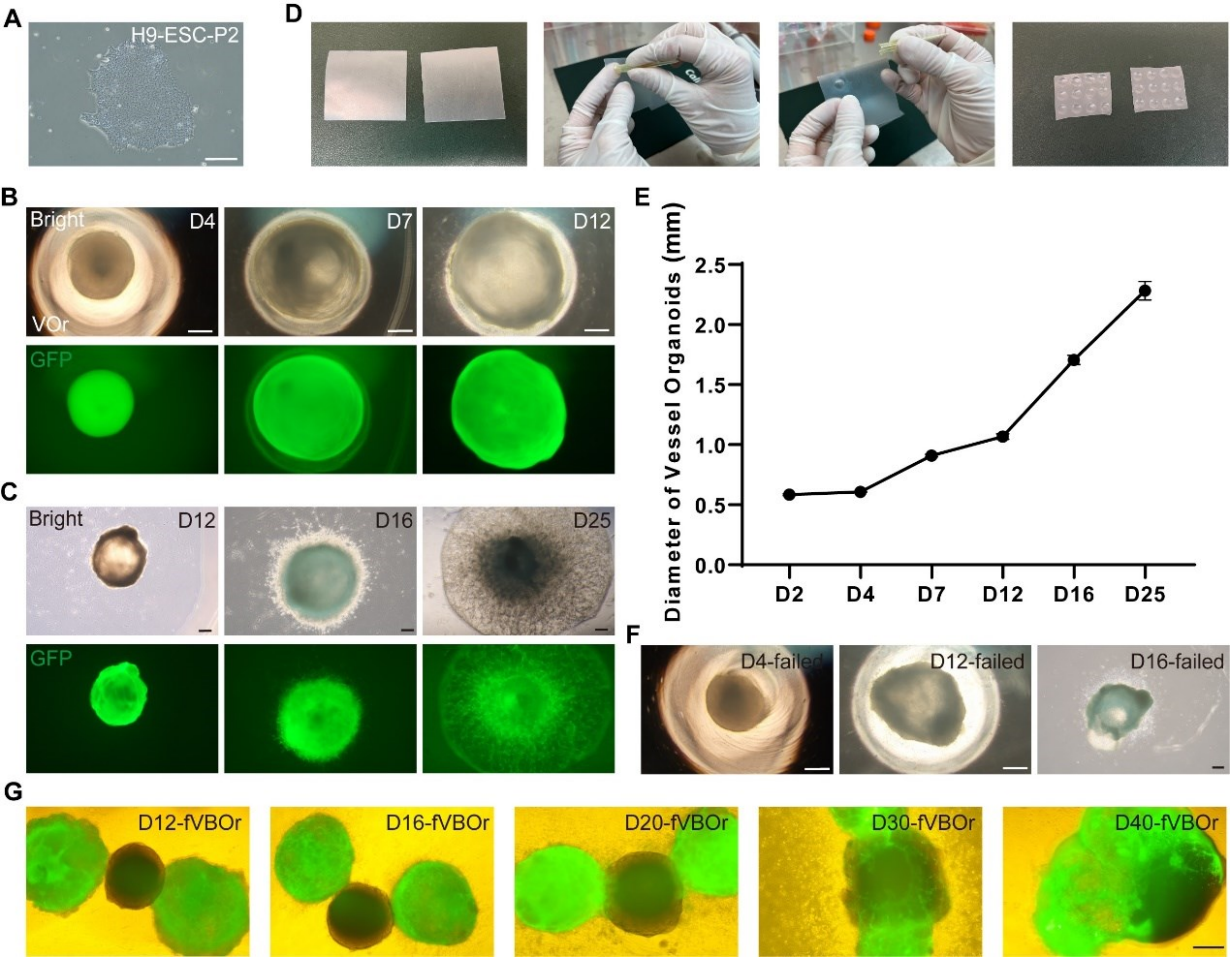
Processes of generating VOrs and fVBOrs and representative images. (A) An H9-ESC colony showing a good stemness state. Scale bar, 200 μm. (B) Developmental stages of VOrs from D4 (day 4) to D12 in a V-bottom 96-well plate. Top, bright field; bottom, GFP. Scale bars, 200 μm. (C) Developmental stages of VOrs from D12 to D25 in dishes. Top, bright field; bottom, GFP. Scale bars, 200 μm. Note the protrusion of vessels to the Matrigel. (D) The processes of making dimpled Parafilm substrate for Matrigel embedding. (E) Quantification of the VOr diameter from D4 to D25. Data are presented as mean ± SEM (15–20 organoids at each time point). (F) The failed cases of VOrs at D4, D12, and D16. Scale bars, 200 μm. (G) The developmental stages of fVBOrs from D12 to D40. GFP, VOrs; Bright, BOrs. VOrs: vessel organoids; BOrs: brain organoids; fVBOrs: fusion vascularized brain organoids. Scale bar, 200 μm.
    - Detach H9-EGFP cells from the Matrigel-coated plate using Accutase to dissociate cell colonies into single cells. Wash the cells with 1× DPBS and add 1 mL of Accutase solution to each well of the 6-well plate. Place cells back in the CO_2_ incubator at 37 °C for 10 min or until the colonies have been dissociated into single cells and then add 2 mL of mTeSR into each well.
*Note: After 5–6 min of digestion, observe the cell states to prevent over-digestion.*
    - Collect the medium into a 15 mL conical tube and centrifuge at 200× *g* for 2 min.
    - Cell counting.
      Remove the supernatant and resuspend the cells in 1 mL of mTeSR (containing 10 μM Y27632). Pipette cell suspension two to three times to avoid the re-aggregation of dissociated single cells and then dilute cells using the fresh aggregation medium at a reasonable ratio for cell counting. Only count the living cells devoid of trypan blue labeling using a hemocytometer or any cell-counting device in the culture room.
*Note: Resuspend the cells evenly before counting, which should be as accurate as possible to reduce the batch-to-batch variation of the initial size of embryoid bodies (EBs). Y27632 is added on the first day of single-cell dissociation and culturing.*
    - Making EBs
      i. Use a V-bottom 96-well plate to form the EBs (early stage of the organoids) from single-cell suspension. Prepare 0.5% Lipidure^®^ solution by dissolving 0.25 g of Lipidure^®^ powder in 50 mL of absolute ethyl alcohol in a sterile 50 mL tube. Add 20 μL of Lipidure^®^ solution into each well of the V-bottom 96-well plate for 10 min, pipette off the remaining liquid, and then leave the plate upright with the lid slightly off in the biosafety cabinet to dry completely for 5 min.
*Note: 20 μL of Lipidure^®^ solution is enough to cover the V-bottom plate. However, more than 20 μL is also acceptable.*
      ii. Dilute stem cells using fresh and warm aggregation medium (see Recipes) to a concentration of 7,000–9,000 cells per 150 μL of medium.
      iii. Plate 150 μL of cells in each well of the Lipidure^®^-coated V-bottom 96-well plate. Place it back in the CO_2_ incubator (37 °C) and culture the cells for 48 h to induce EB formation.
*Note: Generally, 7,000 cells per well are used for generating brain organoids and 9,000 cells per well for generating vessel organoids. From experience, 7,000–9,000 cells per well are conducive to generating the two organoids.*
  2. Organoid induction, days 2–10
    - Day 2: Replace the aggregation medium with 150 μL of mesoderm induction medium (see Recipes) for each well and continue to culture EBs in the CO_2_ incubator at 37 °C for 48 h.
*Note: Tilt the plate and aspirate the aggregation medium with a pipette without disrupting the EBs. Do not handle many organoids simultaneously to prevent them from drying out.*
    - Day 4: Replace the mesoderm induction medium with 200 μL of endothelial induction medium (see Recipes) per well and culture EBs at 37 °C for 72 h.
    - Days 7–11: Replace the endothelial induction medium with 150 μL of endothelial maturation medium (see Recipes) for each well and culture at 37 °C for 48 h. Half-change the endothelial induction medium on days 9 and 11. Endothelial tissues (organoids) have a round and smooth morphology (Figure 2B).
  3. Embedding organoids in Matrigel, day 12
    - Preparing dimpled Parafilm:
      i. Cut the Parafilm into 5 cm × 5 cm squares, layer it on the gloved finger, and then press the broader round end of the 200 μL tips into the Parafilm to create a dimple. Repeat the same action to make a grid of dimples in the Parafilm (Figure 2D).
      ii. Evenly spray 75% alcohol on the two sides of the dimpled Parafilm substrate; then, place the Parafilm in the biosafety cabinet for further sterilization by its own ultraviolet radiation for over 1 h. Place the sterilized Parafilm in sterile 60 mm dishes.
*Note: Any other commercial product can be used to replace the dimpled Parafilm.*
    - Preparing Matrigel.
      Use growth factor-reduced Matrigel to embed organoids. Place the original concentrated Matrigel in the 4 °C fridge one day in advance to enable natural thawing. All Matrigel operations are conducted on ice.
    - Use a disposable Pasteur pipette to individually transfer each vessel organoid to each small dimple in the Parafilm, manually aspirate the extra medium with a 10 μL pipette tip, and then gently drip 10 μL of Matrigel onto each organoid. Gently move the organoid with the tip and adjust its position to the center of the Matrigel droplet.
*Note: Ensure caution to draw as little liquid as possible with the organoids in the dimples and embed with the Matrigel as soon as possible after draining the liquid to avoid the drying out of organoids.*
    - After all the organoids are embedded in the Matrigel, move them into a 37 °C incubator and solidify the Matrigel droplets for over 30 min.
    - Remove the embedded organoids from the Parafilm. Clamp the Parafilm with sterilized tweezers and place in the wells of a 6-well plate or dish with the expansion medium (see Recipes). Make the embedded organoids fall off the Parafilm sheet by gently dripping the medium onto the solidified Matrigel with a 1,000-μL tip.
    - Culture the Matrigel-embedded organoids in a 6-well plate or 60 mm dish for four days with 3 mL or 6 mL of expansion medium per well or dish.
*Note: No more than 10 organoids in a well (6-well plate).*
*Optional step: Manually shake the dish daily to prevent the organoids from sticking to the bottom.*
  4. Vessel organoid maturation, days 16–40 (the morphology is shown in Figure 2C)
    - On day 16, replace the expansion medium with the maturation medium (see Recipes) and move the organoids onto a shaker in the CO_2_ incubator (the shaker speed is 25–30 revolutions per minute).
    - From day 16 onwards, half-change the medium every 2–3 days.
*Note: Due to faster nutrient consumption as reflected by the color change to yellowish after half-changing the medium, the organoids at the late stages can be placed in a T25 flask that contains more culture medium. Please add fresh medium if the old medium evaporates to less than 3 or 6 mL per well or dish.*
- **Brain organoid generation (schematic view shown in Figure 3)**
  **Figure 3.**
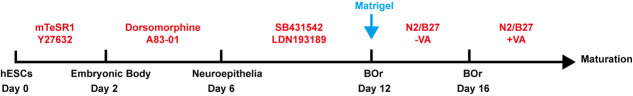
Schematic procedure of generating brain organoids (BOrs) from hESCs
  1. Making EBs, day 0
    - Culture H9 cells as described in section A; when cell density reaches 80% confluence and shows good growth status (flat and well-defined edges of colonies and high nucleus/cytoplasm ratio of cells), the stem cells can be used for organoid generation.
    - Detach H9 cells from the Matrigel-coated plate by adding Accutase solution into the wells. Incubate the cells for 10 min at 37 °C in the CO_2_ incubator and then add 2 mL of mTeSR into the well. Collect the medium into a 15 mL conical tube and centrifuge at 200× *g* for 2 min.
    - Cell counting:
      Remove the supernatant and resuspend the cells with 1 mL of mTeSR containing 10 μM Y27632. Pipette cell suspension two to three times and then dilute cells with fresh aggregation medium at a reasonable ratio for cell counting. Only count live cells devoid of trypan blue labeling.
    - Add 20 μL of Lipidure^®^ solution into each well of a 96-well V-bottom plate for 10 min; aspirate the Lipidure^® ^and dry the plate in the biosafety cabinet for 5 min.
    - Dilute stem cells with the fresh and warm aggregation medium to 7,000–9,000 cells per 150 μL of medium and seed the cells into the V-bottom wells of a 96-well plate. Culture the cells in the CO_2_ incubator at 37 °C for 48 h to form the EBs.
*Note: In general, 7,000 cells per well are used for generating brain organoids.*
  2. Neuroepithelium induction, days 2–10
    - Day 2: Replace the aggregation medium with 150 μL of the ectoderm induction medium (see Recipes) for each well and culture in the CO_2_ incubator at 37 °C for 48 h.
    - Day 4: Half-change the ectoderm induction medium in each well and continue the culturing at 37 °C for an additional 48 h.
    - Day 6: Replace the ectoderm induction medium with 150 μL of neuroepithelium induction medium (see Recipes). After 48 h, half-change the neuroepithelium induction medium on day 8 and then on day 10. Continue culturing at 37 °C for six days.
  3. Embedding neuroepithelial tissues in Matrigel, day 12
    In this step, the neuroepithelial tissues are ready for fusion with the vessel organoids. However, if the researcher wants to separately culture brain organoids, this embedding step is the same as described in step B3.
  4. Brain organoid maturation, days 16–40
    - On day 16, replace the neuroepithelium induction medium with the maturation medium and move the Matrigel droplets to a shaker placed in the CO_2_ incubator (the shaker speed should be 25–30 rotations/min).
    - From day 16 onwards, half-change the medium every 2–3 days.
*Note: Please add fresh medium if the old medium has evaporated to less than 3 or 6 mL per well or dish.*
- **Vascularized brain organoid generation**
  1. Generate vessel organoids by following section B (days 0–10) of this protocol.
  2. Generate neuroepithelial tissues by following section C (days 0–10) of this protocol.
  3. Fusion of the vessel and brain organoids.
    - The preparation steps are the same as those for the culture of vessel organoids on day 12. Gently transfer two vessel organoids and one neuroepithelial tissue one by one to each dimple in the Parafilm; aspirate the excess medium from each tissue and then drip 25 μL of Matrigel onto the tissues.
    - Gently align the three tissues with the neural tissue in the middle of the two vessel organoids using a 10 μL pipette tip. Adjust the position to maintain the three tissues in the same horizontal plane and at the center of the droplet.
  4. Place the droplets with embedded tissues on the Parafilm back into the 37 °C incubator to polymerize the Matrigel for over 40 min. Detach the solidified tissue droplets from the Parafilm as described above and then culture them in a 6-well plate or 60 mm dish for four days in the expansion medium, with each well or dish containing 3 or 6 mL of medium.
*Note: We suggest the use of no more than five fusion tissues in one well. If more than five fusion tissues are cultured, transfer them into a T25 flask using a sterile Pasteur pipette (3 mL) with a broader cutting mouth.*
  5. On day 16, replace the expansion medium with the maturation medium and move the tissues to a shaker placed in the incubator.
  6. As of day 16, half-change the medium every 2–3 days to facilitate the development of the cerebral brain organoid and its vascularization by vessel organoids. Observe the organoid morphology at various stages (Figure 2G).
*Note: Check the color of culture medium and refresh it if it becomes yellow. Please add fresh medium if the old medium has evaporated to less than 3 or 6 mL per well or dish.*

## Data analysis

**Immunofluorescence**

**Figure 4.**
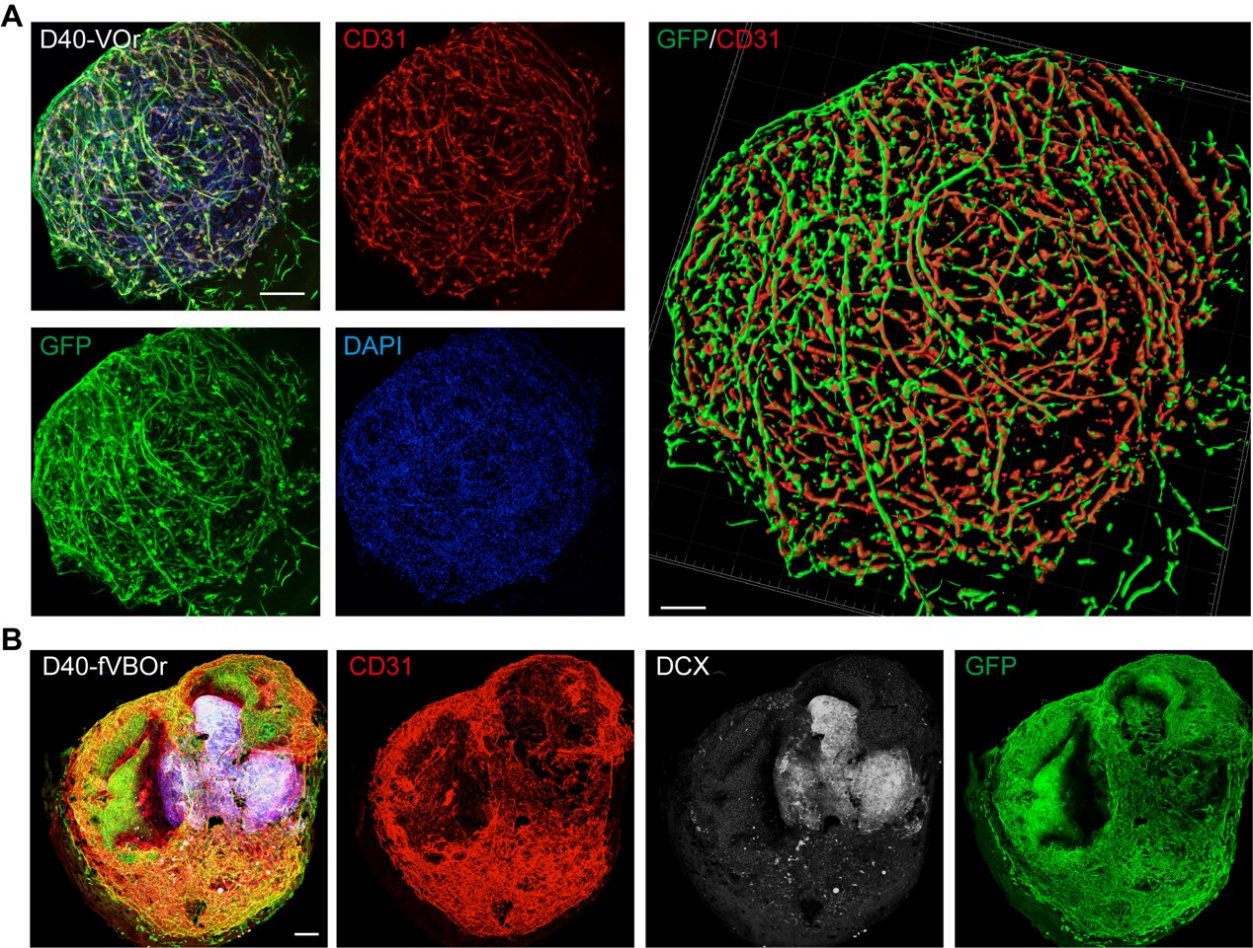
Identification by cell marker staining on vessel organoids (VOrs) and fusion vascularized brain organoids (fVBOrs). (A) Immunostaining of GFP and CD31 in D40 VOrs. Scale bar, 200 μm. Right: Imaris reconstruction of VOrs showing integrated vasculature structures. (B) Immunostaining of CD31 and DCX for labeling vessels and neurons, respectively, in D40 fVBOrs. Scale bar, 200 μm. Images in Figure 4 are quoted from the published article, Figure 1D and Figure 4C (Sun et al., 2022).

**Figure 5.**
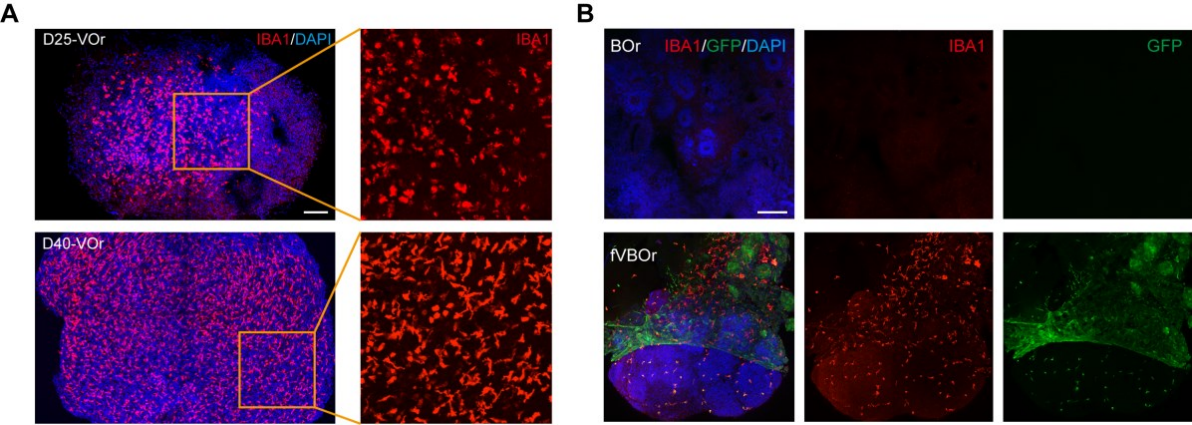
Microglial cells induced in vessel organoids (VOrs) and fusion vascularized brain organoids (fVBOrs). (A) Immunostaining of IBA1 for the labeling of microglial cells in D25 and D40 VOrs. Scale bar, 200 μm. (B) Immunostaining of IBA1 for the labeling of microglial cells in brain organoids (BOrs) and fVBOrs, respectively. Scale bar, 200 μm. Images in Figure 5 are quoted from the published article, Figure 5D and Figure 5F (Sun et al., 2022).

The immunohistochemical images shown in this protocol were generated using whole-mount staining (Figure 4, Figure 5A) or cryo-section staining (Figure 5B). Briefly, the collected organoid samples were fixed in 4% paraformaldehyde (PFA) at 4 °C overnight and then washed three times with DPBS, followed by incubation in 0.5% Triton X-100 at RT (22–25 °C) for 1 h. After blocking with 5% bovine serum albumin (BSA) in 0.1% Triton X-100 at RT for 1 h, organoids were incubated with primary antibodies at 4 °C for over 48 h, washed with DPBS, and then incubated with secondary antibodies at 4 °C for over 48 h. Stained organoids were washed with DPBS three times before confocal imaging. For cryosection staining, the organoids were fixed in 4% PFA at 4 °C overnight and then washed three times with DPBS; then, the samples were dehydrated in 30% sucrose at 4 °C for 24–48 h. Subsequently, the organoids were embedded in OCT compound and cryo-sectioned into 30 μm thick slices. The sectioned slices were boiled in citrate-based antigen retrieval buffer for 10 min, followed by cooling for 60 min. Slices were washed with DPBS three times and incubated in 0.3% Triton X-100 at RT for 30 min, blocked with 5% BSA in 0.1% Triton X-100 at RT for 1 h, and incubated with the primary antibody at 4 °C for over 48 h, followed by washing with DPBS and incubation with the secondary antibody at 4 °C overnight. Secondary antibodies used were Alexa Fluor 488-, 555-, 594-, or 647-conjugated donkey anti-mouse, anti-rabbit, anti-rat, or anti-chicken immunoglobulin G (IgG) respectively (Invitrogen; all used at 1:1,000 dilution). DAPI (2.5 μg/mL) was used to stain the cell nuclei. Primary and secondary antibodies were prepared in DPBS containing 0.1% Triton X-100.

**Analysis of the developmental process**

During culturing, vessel organoids at various developmental stages were imaged from days 2 to 40, as shown in Figures 2B and C. The diameters of the vessel organoids were then quantified (Figure 2E).

As shown in Figure 4A, the vessel organoids exhibited robust vasculogenesis and complex vascular structures expressing the endothelial cell marker CD31. Furthermore, the fusion vascularized brain organoids showed that doublecortin-labeled neurons were surrounded by invaded vessels positively labeled with anti-CD31 antibodies, indicating a connection between neural and blood vessel structures (Figure 4B). Notably, microglial cells were induced in blood vessel organoids (Figure 5A), and these microglial cells invaded the vascularized brain organoids during fusion (Figure 5B). For other experiments on vessel organoids, such as the staining of cell type markers and single-cell analyses, please refer to Figures 1–3 of the published paper (Sun et al., 2022); for the downstream analysis of the fusion vascularized brain organoids, including the blood-brain-barrier, immune functional analyses, and neuro–vascular interactions, please refer to Figures 4–6 of the published paper (Sun et al., 2022).

## Validation of protocol

This protocol has been used and validated in the following research article: Sun et al. (2022). Generation of Vascularized Brain Organoids to Study Neurovascular Interactions. *eLife* 11: e76707. DOI:10.7554/eLife.76707.

## General notes and troubleshooting

Problem 1: Small EBs or scattered cells in V-bottom 96-well plate.

Possible cause: Unhealthy ESCs.

Solution: Culture the stem cells to a good state for generating organoids. (Good state means: high nucleus/cytoplasm ratio of cells, flat and well-defined edge of colonies, and approximately 80% cell confluent.)

Problem 1: Small EBs or scattered cells in V-bottom 96-well plate.

Possible cause: Insufficient cells or starting cells with an extensive number of dead cells.

Solution: Calculate the cells accurately with trypan blue.

Problem 1: Small EBs or scattered cells in V-bottom 96-well plate.

Possible cause: Omitted treatment of the wells with Lipidure^®^ solution.

Solution: Treat the wells with Lipidure^®^ solution.

Problem 2: The VOrs detached from the Matrigel when embedding.

Possible cause: Insufficient time for Matrigel solidification.

Solution: Prolong the time for Matrigel solidification in a 37 °C incubator.

Problem 3: Separation of organoid and Matrigel after embedding.

Possible cause: Strong pipetting during the medium change.

Solution: Gently add fresh medium along the wall of the well or dish; avoid touching the Matrigel with tips during medium renewal.

Problem 3: Separation of organoid and Matrigel after embedding.

Possible cause: Fast shaking speed.

Solution: Check the shaking speed daily and keep the shaker at a low speed.

Problem 4: VOrs show a bad state without strong vasculogenesis during the maturation stage.

Possible cause: The VOrs stick to the bottom of the dishes.

Solution: Put the VOrs in a rotating shaker to avoid sticking.
